# Epigenetic Regulation of Genomic Stability by Vitamin C

**DOI:** 10.3389/fgene.2021.675780

**Published:** 2021-05-04

**Authors:** John P. Brabson, Tiffany Leesang, Sofia Mohammad, Luisa Cimmino

**Affiliations:** ^1^Department of Biochemistry and Molecular Biology, Miller School of Medicine, University of Miami, Miami, FL, United States; ^2^Sylvester Comprehensive Cancer Center, Miller School of Medicine, University of Miami, Miami, FL, United States

**Keywords:** DNA methylation (5mC), DNMT, 5-hydroxymethylation (5hmC), TET, genomic stability, vitamin C

## Abstract

DNA methylation plays an important role in the maintenance of genomic stability. Ten-eleven translocation proteins (TETs) are a family of iron (Fe^2+^) and α-KG -dependent dioxygenases that regulate DNA methylation levels by oxidizing 5-methylcystosine (5mC) to generate 5-hydroxymethylcytosine (5hmC), 5-formylcytosine (5fC), and 5-carboxylcytosine (5caC). These oxidized methylcytosines promote passive demethylation upon DNA replication, or active DNA demethylation, by triggering base excision repair and replacement of 5fC and 5caC with an unmethylated cytosine. Several studies over the last decade have shown that loss of TET function leads to DNA hypermethylation and increased genomic instability. Vitamin C, a cofactor of TET enzymes, increases 5hmC formation and promotes DNA demethylation, suggesting that this essential vitamin, in addition to its antioxidant properties, can also directly influence genomic stability. This review will highlight the functional role of DNA methylation, TET activity and vitamin C, in the crosstalk between DNA methylation and DNA repair.

## Introduction

The protective role of vitamin C in cancer progression has historically been attributed to its antioxidant activity and the prevention of DNA damage induced by oxidative stress (Padayatty and Levine, 2016). Vitamin C also enhances the activity of a large family of iron (Fe^2+^) and α-ketoglutarate-dependent dioxygenases (α-KGDDs), which include epigenetic regulators of DNA methylation known to play important roles in the maintenance of genomic stability. Ten-Eleven Translocation proteins (TETs) are a subfamily of α-KGDDs that promote DNA demethylation in the genome (Tahiliani et al., 2009). Loss of function in TET proteins and altered levels of DNA methylation are hallmarks of cancer (Figueroa et al., 2010a, b; Akalin et al., 2012) that drive genomic instability and malignant transformation (An et al., 2015; Ko et al., 2015). Recent studies have shown that vitamin C, by enhancing TET activity, can directly influence DNA methylation levels that in turn alter chromatin structure, and the expression of tumor suppressors and DNA repair enzymes. Vitamin C deficiency has been widely reported in cancer patients (Mayland et al., 2005; Huijskens et al., 2016) and accelerates cancer progression in disease models (Agathocleous et al., 2017). In addition to its potential role in the prevention of cancer, vitamin C added to cell culture media can improve the quality of stem cells and reprogrammed cells for use in stem cell therapies and regenerative medicine by maintaining genomic integrity (Li et al., 2009; Wang et al., 2011; Chen J. et al., 2013; Gustafson et al., 2015; Hore et al., 2016). Through its ability to promote DNA demethylation, vitamin C has clinical application in the treatment of cancer and especially hematological malignancies where mutation in TET enzymes and aberrant DNA methylation are frequently observed. While several recent reviews have underscored the role of vitamin C as an anticancer agent (Das et al., 2021), modulator of immune responses (Yue and Rao, 2020), and in the reprogramming of stem cells (Lee Chong et al., 2019), herein we highlight these activities within a context of DNA damage, repair, and genomic stability. Understanding how DNA methylation levels and TET activity influence genomic stability provides the context in which vitamin C, as a co-factor of TET enzymes, can play a pivotal role as an epigenetic regulator of DNA damage and repair.

## The Role of DNA Methylation in the Maintenance of Genomic Stability

DNA methylation directly regulates essential biological functions such as gene expression, chromatin organization, DNA imprinting and X-chromosome inactivation that in combination instruct embryonic development and cellular differentiation (Guo et al., 2014). DNA methylation also plays direct and indirect roles in maintaining genomic stability. Both hypomethylation and hypermethylation of DNA is associated with increased genomic instability that can lead to malignant transformation. Under normal, steady-state conditions, up to 80% of cytosines, in the context of CpG dinucleotides, are methylated in the mammalian genome, reviewed in Law and Jacobsen (2010). DNA methylation is conventionally known as a repressive epigenetic mark, and the majority of methylated cytosines are concentrated in heterochromatic regions, ensuring that chromatin remains closed and genes silenced when not required to be actively transcribed by the cell. DNA methylation also directly silences gene expression by hypermethylation of CpG-rich regions known as CpG islands (CGIs) in promoter regions that alter the ability to recruit transcription factors (Curradi et al., 2002). This is a key mechanism by which DNA methylation regulates gene expression, as approximately 70% of human gene promoters have associated CGIs (Saxonov et al., 2006; Deaton and Bird, 2011). Furthermore, almost 50% of the human genome consists of long terminal repeats (LTRs), short or long interspersed nuclear elements (SINES or LINES), and other endogenous retroviruses that are enriched for CpGs and silenced into heterochromatic regions by both DNA methylation and repressive histone modifications (Kondo and Issa, 2003; Pehrsson et al., 2019). DNA hypomethylation at repetitive regions in the genome can reduce the formation of heterochromatin, leading to transposition of DNA and the aberrant expression of oncogenes that can drive tumorigenesis (Lopez-Moyado et al., 2019). DNA hypermethylation can also promote genomic instability, by silencing the expression of DNA repair genes, or by inhibiting the recruitment of DNA repair proteins (Toffolatti et al., 2014; Tsuboi et al., 2020). Methylated cytosines are also intrinsically more mutagenic than unmethylated cytosines (Poulos et al., 2017; Kusmartsev et al., 2020). Balancing the activity of writers and erasers of DNA methylation ensures that epigenetic information is interpreted and inherited correctly, but also protects cells from acquiring permanent changes to the genetic code.

## Writers and Erasers of DNA Methylation

DNA methyltransferases (DNMT1, 3A and 3B) catalyze the transfer of a methyl group from *S*-adenosyl-L-methionine (SAM) to the 5′ position of cytosine residues generating 5-methylcytosine (5mC) in the genome. DNMT1 is essential for the maintenance of methylation marks during DNA replication, whereas DNMT3A/B are responsible for *de novo* synthesis and their activity is independent of the cell cycle (Bestor et al., 1988; Okano et al., 1999). DNMT1 preferentially recognizes hemi-methylated DNA so that DNA methylation patterns are inherited upon DNA replication (Bashtrykov et al., 2012), whereas DNMT3A and B show an equivalent affinity for both hemimethylated and unmethylated DNA (Kareta et al., 2006).

TET proteins (TET1-3) are a sub-family of α-KGDDs that promote DNA demethylation by catalyzing the iterative oxidation of 5mC to generate 5-hydroxymethylcytosine (5hmC), 5-formylcytosine (5fC), and 5-carboxylcytosine (5caC) (Tahiliani et al., 2009; He et al., 2011; Ito et al., 2011). 5hmC is a stable modification that constitutes 5–10% of the total level of 5mC in embryonic stem cells (ESCs) (Tahiliani et al., 2009) but this frequency can vary widely in different adult tissues, with 5hmC present at 40% the level of 5mC in Purkinje cells of the brain (Kriaucionis and Heintz, 2009) compared to 1% in immune cells (Ko et al., 2010). The presence of 5hmC in the genome causes the passive loss of DNA methylation upon DNA replication, given that DNMT1 is unable to recognize hemi-methylated 5hmC sites (Otani et al., 2013). 5fC and 5caC levels are rare modifications in the genome and constitute approximately 2 or 0.5% of the total level of 5hmC in wild-type mouse ESCs, respectively (He et al., 2011; Ito et al., 2011).

The low abundance of 5fC and 5caC in DNA is attributed to their removal and replacement with an unmethylated cytosine by active DNA demethylation via base excision repair (BER). The conversion of 5hmC to 5-hydroxymethyluracil (5hmU) by cytidine deaminase (AID or APOBEC) is one proposed mechanism by which thymine or uracil DNA glycosylases (TDG or SMUG1, respectively) excise oxidized mCs (Cortellino et al., 2011). Subsequently it was shown that TDG most likely targets 5fC:G and 5caC:G mismatches for removal, given that TDG-deficient ESCs accumulate up to 10-fold higher levels of 5fC and 5caC in their genome (He et al., 2011; Shen et al., 2013; Song et al., 2013), and TDG targets 5caC:G and 5fC:G with higher affinity than T:G with no activity toward 5hmC:G (Maiti and Drohat, 2011; Zhang et al., 2012). Other components of the DNA damage and BER machinery have been identified as specific readers of 5fC or 5caC, including p53, TDG, PARP, GADD45 and NEIL1/2 (Spruijt et al., 2013), and depletion studies of these factors in cells have shown that their activity is required to prevent DNA hypermethylation (Cortellino et al., 2011; Ciccarone et al., 2012; Li et al., 2015; Schomacher et al., 2016; Tovy et al., 2017). TET enzymes have also been shown to oxidize thymine to generate 5hmU directly, leading to 5hmU:A mismatches (Pfaffeneder et al., 2014; Olinski et al., 2016). Protein readers of 5hmU:G and 5hmU:A also include chromatin regulators and DNA repair enzymes involved in BER (Pfaffeneder et al., 2014), and the repair of 5hmU:A by long-patch BER or non-canonical mismatch repair could lead to the indirect removal of adjacent 5mC in the genome (Metivier et al., 2008; Santos et al., 2013; Grin and Ishchenko, 2016; Spada et al., 2020). In primed embryonic murine pluripotent stems cells (PSCs) and the developing zygote, other non-oxidative and cell-cycle independent mechanisms of DNA demethylation have also been reported that may utilize deamination of mC by AID and DNA repair processes (Santos et al., 2013; Amouroux et al., 2016; Spada et al., 2020), however, the majority of active DNA demethylation in mPSCs was shown to be driven by oxidation of 5mC (Spada et al., 2020). These studies provide further evidence that TET-oxidized bases and DNA repair mechanisms can work together, directly or indirectly, to mediate DNA demethylation in the genome.

Studies into the genomic distribution of 5hmC, 5fC, and 5caC revealed their enrichment in correlation with active gene expression at transcriptional start sites and gene bodies (Ficz et al., 2011; Williams et al., 2011; Wu H. et al., 2011; Raiber et al., 2012; Song et al., 2013; Neri et al., 2015), enhancers (Lu et al., 2014; Rasmussen et al., 2015), the edges of large “canyons” of low DNA methylation in the genome (Jeong et al., 2014), and at the boundaries of topologically associated domains (TADs) marked by the insulator protein CCCTF binding factor (CTCF) (Song et al., 2013; Nanan et al., 2019). Several laboratories have developed methods to sequence the genomic distribution of 5hmU at base resolution (Yu et al., 2015; Bullard et al., 2017; Kawasaki et al., 2017), however, the patterning of this modification in the genome of mammalian cells has not yet been described.

## Aberrant DNA Methylation Drives Genomic Instability and Cancer Progression

**FIGURE 1:**
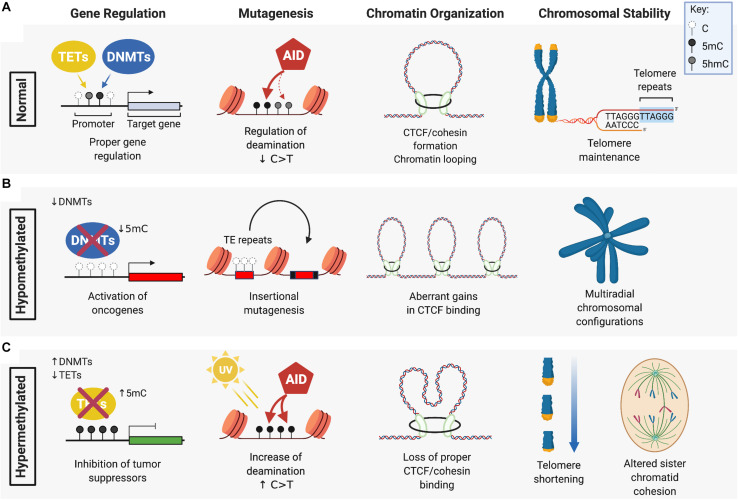
The role of DNMTs and TETs in DNA methylation maintenance and genomic stability. **(A)** DNA methyltransferases (DMNTs) regulate the methylation of cytosine residues (white) in CpG islands typically found in the promoter region of target genes, while Ten-Eleven Translocation (TET) enzymes promote the demethylation of 5-methylcytosines (5mC) (black). Together, these enzymes regulate normal DNA methylation patterns and genomic stability by ensuring: proper silencing and activation of gene expression; cytosine deamination by AID or APOBEC (not shown) that preferentially target 5mC over C/5hmC (gray) for C > T transition; CTCF/cohesin-guided boundaries between enhancers and promoters of genes; and maintenance of telomeres. **(B)** DNA hypomethylation, caused by a loss of function of DNMTs, can lead to: aberrant oncogene activation by removal of promoter silencing; upregulated activity of transposable elements (TE) that drive insertional mutagenesis; gains in CTCF binding that exacerbate oncogene expression; and loss of heterochromatin at peri-centromeric regions causing defective chromosome segregation and multi-radial configurations (specifically seen in ICF patients with DNMT3B mutation). **(C)** DNA hypermethylation is associated with: aberrant silencing of tumor suppressors from increased DNMT activity or loss of TET function; an increased frequency of C > T transitions due to enhanced mutagenicity and deamination of 5mC; loss of CTCF/cohesin binding leading to gene mis-regulation; and telomere shortening and altered sister chromatid cohesion. Figure created with BioRender.com.

Differences in the level of DNA methylation and oxidized 5mC, or their aberrant distribution across the genome, can influence how cells interpret these epigenetic cues. Both hypomethylation and hypermethylation of the DNA is associated with increased genomic instability that can lead to malignant transformation (Figure 1). Dysregulation of DNA methylation patterning can occur by several mechanisms including defective or decreased activity of DNMT or TET DNA demethylases that leads to hypomethylation and/or hypermethylation. DNA methylation changes, in combination with altered expression or activity of cytosine deaminases, and other readers or repair enzymes recruited at 5mC, 5hmC, 5fC, or 5caC modified cytosines, can accelerate mutational processes that drive cancer progression.

## DNA Hypomethylation and Genomic Instability

### Upregulation of Oncogene Expression

Defects in DNMT expression and function can cause global hypomethylation that correlates with genomic instability and tumor progression due to loss of silencing at the loci of oncogenes (Soes et al., 2014; Kushwaha et al., 2016; Sheaffer et al., 2016). One of the most frequently observed upregulated oncogenes in cancer that exhibits a hypomethylated gene locus and is known to cause genomic instability is the transcription factor c-Myc. Large genomic amplifications induced by c-Myc overexpression are attributed to replicative stress caused by unscheduled DNA replication and increased oxidative stress leading to DNA breaks (Dominguez-Sola et al., 2007; Kuzyk and Mai, 2014). While c-Myc DNA hypomethylation is observed in many different cancers, other examples of hypomethylated oncogenic loci are cancer-specific, such as the melanoma-associated antigens (MAGE) gene that is hypomethylated specifically in gastric cancer and colorectal cancer (Honda et al., 2004; Kim et al., 2006) or synuclein γ (SNCG) in breast and ovarian cancers and solid tumors of the liver, gastric and (Gupta et al., 2003; Liu et al., 2005). Overexpression of these oncogenes promote, proliferation, metastasis, disruption of mitotic checkpoints, enhanced transcriptional activity and accelerated rates of chromosomal instability.

### Increased Insertional Mutagenesis and Chromosomal Anomalies

Hypomethylated repetitive DNA sequences, including CG repeats in CGIs and in transposable elements (TEs), are commonly found in cancers that could be caused by loss of function in DNA methyltransferase activity. Hypomorphic activity of *Dnmt1* in mice induces thymic lymphomas with recurring insertions of a transposable element within the Notch gene leading to its oncogenic activation (Howard et al., 2008). LINE-1 and Alu elements are retrotransposons that constitute over 30% of the human genome and are normally inhibited by DNA methylation yet become frequently hypomethylated in cancer (Daskalos et al., 2009; Pehrsson et al., 2019). Increased LINE-1 and Alu activity causes genomic instability by promoting deletions, chromosome breaks, and translocations (Mandal et al., 2013; Bakshi et al., 2016).

DNA hypomethylation is also observed at microsatellite and pericentromeric heterochromatic regions in chromosomes of *DNMT3B*-mutant disease. *DNMT3B* loss of function mutations have been described in ∼60% of patients with an autosomal recessive disorder known as immunodeficiency, centromeric instability and facial anomaly (ICF) syndrome (Hansen et al., 1999; Okano et al., 1999; Xu et al., 1999). ICF is a rare disease showing symptoms in early childhood of recurrent gastrointestinal and pulmonary infections as a result of agammaglobulinemia (Ehrlich et al., 2008; Hagleitner et al., 2008). ICF patients display evident decondensation of heterochromatin caused by DNA hypomethylation at pericentromeric regions in chromosomes 1, 9, and 16, causing upregulated gene expression at these loci, chromosome breaks, and rearrangements in radial structures that are detectable in stimulated lymphocytes and increase the risk of hematological malignancies (Brown et al., 1995; Kaya et al., 2011; Gossling et al., 2017).

### Loss of Chromatin Organization

Cancer cells on average display up to 30% genome-wide losses in DNA methylation (Ehrlich and Lacey, 2013; Baylin and Jones, 2016) which could significantly impact three-dimensional chromatin architecture and genomic stability by altering oncogene activity and activation of TEs. CTCF, in cooperation with cohesin proteins, regulates long-range looping interactions between enhancers and promoters (Dixon et al., 2012) and preferentially binds at hypomethylated CpGs of *cis*-regulatory insulator regions of the genome (Nanavaty et al., 2020). DNA hypomethylation could allow for aberrant gains in CTCF binding, creating stronger insulation at oncogenic super-enhancers and an increased frequency of tandem duplications (Gong et al., 2018). Mis-regulated gene expression via stronger TAD boundaries could also cooperate with oncogenic transcription factors to exacerbate genomic instability (Fang et al., 2020). Thus, hypomethylated states may confer an advantage of cancer cells by the increase and maintenance of elevated oncogenic expression during tumor development (Kang et al., 2015).

## DNA Hypermethylation (Or Loss of Hydroxymethylation) and Genomic Stability

### Silencing of Tumor Suppressors

Hypermethylation of the genome is associated with aberrant silencing of many tumor suppressors that could drive or accelerate carcinogenesis. DNA hypermethylation can be caused by a decrease in the activity of DNA demethylases such as the TET proteins leading to the silencing of DNA repair enzymes and tumor suppressors. Silencing due to aberrant DNA hypermethylation at imprinted loci (Rainier et al., 1993; Moulton et al., 1994; Steenman et al., 1994; Plass and Soloway, 2002), senescence genes (Kamb et al., 1994; Zhao et al., 2016; Xie et al., 2019) and lineage specific transcription factors, that normally slow proliferation and drive differentiation, reviewed in (Suelves et al., 2016; Pfeifer, 2018), may cause a buildup in unchecked DNA damage and DNA replication errors.

### Increased Methyl-Cytosine Mutagenicity

Methylated cytosines are approximately fivefold more likely to undergo mutagenesis than unmethylated cytosines (Poulos et al., 2017). This could be due to a greater tendency for 5mC to spontaneously deaminate compared to unmethylated cytosines (Supek et al., 2014; Blokzijl et al., 2016). Furthermore, C > T and G > A mutations disproportionally affect CpG dinucleotides. In one study, approximately 18% of C > T and G > A missense and nonsense mutations in genes of inherited human diseases occurred within CpGs, a frequency 10-fold higher would have been expected by chance alone (Cooper et al., 2010). Cytosine deaminases (AID and APOBEC1-3) also exhibit greater activity toward 5mC than 5hmC (Nabel et al., 2012; Rangam et al., 2012), and 5mC deamination generates T:G mismatches that can potentially recruit error-prone mismatch repair (MMR) complexes (Cortellino et al., 2011). Importantly, C > T mutation rates are 50% lower for C or 5hmC compared to 5mC (Tomkova et al., 2016). Given that C > T transition mutations are the most frequent, age-associated mutation signature in cancer (Alexandrov et al., 2013; Kandoth et al., 2013) loss of TET activity could mimic a premature aging phenotype with regard to the frequency at which C > T mutations accumulate. Distinct mutational signatures are also caused by aberrant deaminase activity, with AID/APOBEC-driven signatures dominated by high levels of C > T transitions in specific cancers (Alexandrov et al., 2020). Upregulated oncogenic activity of AID/APOBEC deaminases, in combination with elevated levels of 5mC in the genome, could synergistically accelerate malignant transformation.

### Loss of Telomere Maintenance, Altered Chromatin Boundaries and Chromosomal Instability

Studies in ESCs have shown that loss of TET function causes decreased 5hmC and DNA hypermethylation in telomeric regions, that leads to telomere shortening, reduced telomere recombination and chromosome segregation defects (Lu et al., 2014; Kafer et al., 2016; Yang et al., 2016). Sub-telomeres are also hypermethylated in TET depleted ESCs, which may further impede telomere elongation by recombination (Yang et al., 2016). DNA hypermethylation caused by loss of TET activity increases nucleosome occupancy, and subsequently, loss of CTCF binding and a block in the downstream recruitment of the cohesin complex, reducing the formation of CTCF/cohesion-mediated chromatin loops thereby downregulating the expression of neighboring genes (Wiehle et al., 2019; Nanavaty et al., 2020). Loss of CTCF itself can also induce DNA hypermethylation at CTCF binding sites and the fusion of TAD boundaries that establish oncogenic expression patterns and increased cancer progression (Akdemir et al., 2020; Damaschke et al., 2020). TETs also regulate chromosomal architecture by protecting large undermethylated genomic regions known as DNA methylation “canyons” from becoming hypermethylated (Wiehle et al., 2016; Zhang X. et al., 2016). Loss of *Dnmt3a* or *Tet2* causes canyon edges to collapse and become hypermethylated, suggesting that de novo methyltransferase activity and Tet2-mediated hydroxylation of 5mC work together to maintain 5hmC and hypomethylation at these loci (Jeong et al., 2014; Zhang X. et al., 2016).

Studies using ESCs with a triple knockout of all three TET enzymes revealed an increase of 5mC signal across all chromosomes by whole-genome bisulfite sequencing (Lu et al., 2014) and an increased frequency in telomere loss and chromosomal fusion (Yang et al., 2016). Genomic instability has also been observed in *Tet2/3* double knockout hematopoietic stem and progenitor cells (HSPCs) and immature myeloid cells, which show impairment of DNA repair responses, lower homologous recombination (HR) or non-homologous end-joining (NHEJ) gene expression and spontaneously accumulate DNA double strand breaks (DSBs) marked by increased phosphorylated histone H2A.X (γH2AX) in comparison to WT cells (An et al., 2015). These studies, in combination with numerous reports of TET deficiency in other solid tumors (Haffner et al., 2011; Kudo et al., 2012; Yang et al., 2013; Munari et al., 2016) demonstrate the loss of 5hmC by defective TET enzymatic activity as a hallmark of cancer that may drive genomic instability.

## DNA Damage Induces Changes in DNA Methylation and 5hmC Formation

DNA methylation and oxidized mCs regulate the susceptibility of cells to genomic instability by also actively participating in sensing and repair processes upon DNA damage. C > T transition mutations could lead to losses in methylation at otherwise silenced gene loci that accumulate over our lifetime due to spontaneous deamination and exposure to ionizing radiation or chemical carcinogens (Baylin and Jones, 2016). UV irradiation primarily induces C > T transitions in the epidermis, with cells exposed to UVA biased toward mutations of CC > TT dipyrimidines more frequently than by UVB, and with a greater propensity for 5mC than C (Martinez-Fernandez et al., 2017; Ikehata, 2018). UV irradiation can also induce the expression of DNMT1 in human dermal fibroblasts leading to DNA hypermethylation at specific gene loci, such as TIMP2 (Kim et al., 2018).

Reactive oxygen species (ROS) are estimated to create up to 50,000 DNA lesions in a single human cell, which can lead to potentially oncogenic mutations if not repaired (Rossi et al., 2007). ROS can influence DNA methylation levels by the oxidation of guanosine to 8-oxo-2′-deoxyguanosine (8-oxo-dG) or by hydroxyl radicals that via abstraction of a hydrogen from the methyl group of mC leads to the formation of 5hmC (Madugundu et al., 2014). The presence of 8-oxo-dG can lead to DNA hypomethylation, by preventing the adjacent cytosine in a Cp8-oxo-dG dinucleotide from being recognized by DNMTs, thereby promoting passive DNA demethylation. Furthermore, 8-oxo-dG DNA glycosylase (OGG1) recruited to these lesions interacts with TET1 that can trigger active DNA demethylation by BER (Zhou et al., 2016). ROS can also induce G quadruplex formation as well as R-loops (DNA-RNA hybrids that are enriched at CpG islands), which can cause genomic instability at active transcriptional sites and delay the removal and repair of these ROS-induced damaged bases (Tan et al., 2020). Further direct evidence of the involvement of TET enzymes in DNA repair come from studies in ESCs, where genotoxic insults such as aphidicolin treatment to induce double strand breaks (DSBs) were shown to induce 5hmC, but not 5mC, foci that co-localized with γH2AX marks, and the repair proteins 53BP1 and RAD51 (Kafer et al., 2016). Together these studies illustrate how TETs can act as the bridge between DNA methylation regulation and DNA repair. The ability of TET proteins to influence transcription and DNA repair at oxidized mCs implies a dual role in the regulation of gene expression and DNA damage responses.

## DNMT and TET Loss of Function in Cancer

The strong association between aberrant DNA methylation and tumorigenesis is evidenced by the frequent occurrence of mutations, and altered expression levels, of DNMT and TET enzymes in solid tumors and hematological malignancies. *DNMT1* and *DNMT3B* mutations or amplifications are more common in cancers of epithelial tissues of the breast, ovaries, skin, bladder, lung and colon (reviewed in Zhang et al., 2020), whereas loss-of-function *DNMT3A* mutations are the most frequent lesions identified in acute myeloid leukemia (AML) where they are found on average in 30% of patients (Ley et al., 2010; Cancer Genome Atlas Research, 2013; Brunetti et al., 2017). The mutation hotspot encoding arginine 882 (R882) accounts for ∼65% of all *DNTM3A* variants and blocks its methyltransferase activity (Russler-Germain et al., 2014; Nguyen et al., 2019; Gao et al., 2020) leading to genome-wide DNA hypomethylation with focal increases in promoter CGI DNA methylation that are hallmarks of AML (Figueroa et al., 2010b; Spencer et al., 2017). *DNMT3A* mutations also predispose patients with pre-malignant clonal hematopoiesis to transformation and drive relapse in AML patients (Jaiswal et al., 2014; Buscarlet et al., 2017). The inability of DNMT3A R882 mutants to sense and repair DNA torsional stress results in increased mutagenesis and resistance to anthracycline (Guryanova et al., 2016) and demonstrates how the role of these epigenetic regulators in DNA damage-sensing, in combination with altered genomic methylation, can drive cancer progression.

Unopposed DNA methyltransferase activity due to loss of TET function can cause DNA hypermethylation, and decreased *TET1-3* expression or reduced 5hmC levels in the genome are common across multiple solid tumors (Haffner et al., 2011; Kudo et al., 2012; Yang et al., 2013, 2015) and blood cell malignancies (Ko et al., 2010; Lemonnier et al., 2018; Zhang et al., 2018). Similar to the lineage bias observed for mutation of *DNMTs*, *TET1* and *TET3* are more frequently mutated in carcinomas (Li L. et al., 2016; Lee et al., 2020), whereas *TET2* deletions and missense mutations induce loss of its catalytic function and are observed at much higher frequency, in ∼10-30% of patients with either clonal hematopoiesis ((Busque et al., 2012; Jaiswal et al., 2014; Bowman et al., 2018), myeloid (Abdel-Wahab et al., 2009; Delhommeau et al., 2009; Langemeijer et al., 2009), or lymphoid (Quivoron et al., 2011; Lemonnier et al., 2012) malignancies.

*DNMT3A* and *TET2* mutation are independently associated with an adverse outcome and poor prognosis in intermediate risk AML (Kosmider et al., 2009; Ribeiro et al., 2012). Furthermore, the prevalence of *DNMT3A* and *TET2* mutation in hematological malignancies, and their early emergence from within the HSPC compartment to drive transformation (Delhommeau et al., 2009; Quivoron et al., 2011; Welch et al., 2012; Papaemmanuil et al., 2013), emphasizes the importance of DNA methylation in the pre-malignant regulation of stem cell self-renewal and blood cell lineage differentiation. Hypermethylated CpGs that should normally be targeted by TET2 for demethylation could suffer increased C > T transition rates, based on the higher mutagenicity of 5mC compared to unmethylated cytosines or 5hmC (Poulos et al., 2017). Cells from myelodysplastic (MDS) and AML patients with *TET2* mutation harbor more non-synonymous somatic mutations than *TET2* wild-type patients, and HSPCs of *Tet2* knockout mice also exhibit increased mutation rates (Pan et al., 2017). Whether CGI hypermethylation in clonal hematopoiesis patients increases susceptibility to additional mutations that drive transformation is not yet known. Both DNMT3A and TET2 have been shown to prevent DNA hypermethylation of canyons and promoter CGIs, suggesting that *de novo* methylation and 5hmC formation by DNMT3A and TET2 work simultaneously in HSPCs to prevent specific tumor suppressor gene loci from becoming dysregulated, and their potential cooperation at these sites requires further study.

## Vitamin C Is a Co-Factor of TET Enzymes and Promotes DNA Demethylation

**FIGURE 2:**
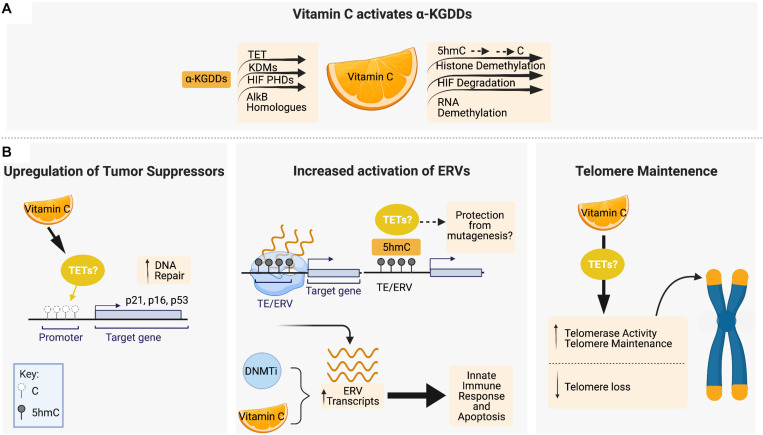
Vitamin C maintains genomic stability through interactions with epigenetic regulators, tumor suppressor upregulation and telomere maintenance. **(A)** Vitamin C serves as a cofactor for members of the α-ketoglutarate-dependent dioxygenases (α-KGDD) family such as TETs, KDMs, HIF PHDs, and ALKBHs, which can work to maintain genomic stability through the demethylation of 5mC residues, histone demethylation, HIF-1α degradation, and RNA demethylation, respectively. **(B)** Vitamin C supplementation has been shown to promote DNA demethylation in the promotor regions of tumor suppressor loci encoding p16, p21, and p53. Vitamin C promotes DNA demethylation and 5hmC formation at transposable elements (TEs) in the genome, leading to upregulated expression of endogenous retroviral genes (ERVs) in combination with DNA methyltransferase inhibitors (DNMTis). In cancer cells, ERV upregulation initiates an innate immune response and apoptotic cell death which is enhanced by vitamin C. Vitamin C treatment has also been shown to increase telomerase activity and the expression of genes that protect telomere integrity and decrease the rate of telomere loss. Figure created with BioRender.com.

The recent discovery that vitamin C can act as an epigenetic regulator by enhancing the activity of α-KGDDs such as the TET proteins has transformed our understanding of the role of vitamin C in biology (Figure 2A). Removal of histone and DNA methylation is essential for the efficient epigenetic reprogramming and generation of induced pluripotent stem cells (iPSCs) from somatic cells. Studies using ESCs and reprogramming fibroblasts were the first to show that vitamin C, in a TET-dependent manner, could increase 5hmC, 5fC, and 5caC production, triggering a global DNA hypomethylation that improved the quality of cells in culture and enhance the formation of iPSCs (Blaschke et al., 2013; Chen Q. et al., 2013; Minor et al., 2013; Yin et al., 2013). ESCs express high levels of TET1 and TET2 (Koh et al., 2011; Dawlaty et al., 2013), and 100 μM vitamin C is sufficient to increase 5hmC by up to ∼4-fold above basal levels in ESCs within 24hrs of treatment, with even larger effects on the levels of 5fC (10-fold increase) and 5caC (20-fold increase) (Yin et al., 2013). A study using human colorectal cancer cells also reported significantly elevated levels of 5hmU (up 18.5-fold increase) in response to vitamin C treatment (Modrzejewska et al., 2016). In HSPCs and human leukemia cell lines, treatment with low or high doses of vitamin C also cause 2–4 fold increases in 5hmC formation and genome-wide DNA hypomethylation (Liu et al., 2016; Cimmino et al., 2017; Mingay et al., 2018) similar to what has been observed in ESCs (Chung et al., 2010). Subsequent studies of vitamin C treatment in other tissue specific stem cells (He et al., 2015; Wulansari et al., 2017) and carcinoma cells of the kidney, bladder, lung, colon and breast, amongst others (Ge et al., 2018; Peng et al., 2018; Sant et al., 2018) all reported similar increases in 5hmC formation and/or DNA hypomethylation.

### Effects of Vitamin C-Mediated 5hmC Formation and DNA Demethylation

The effect of vitamin C treatment on cell viability and genomic stability under steady-state conditions or in response to DNA damage can vary depending on developmental stage or normal versus malignant cell context. Vitamin C treatment can promote survival and maintain proliferation while protecting stem cells from DNA damage and senescence, whereas in cancer cells, the reactivation of tumor suppressors can reduce cell viability and alter sensitivity to therapeutic agents. Given that TET enzymes play a primary role as tumor suppressors, the ability to enhance 5hmC formation and DNA hypomethylation by vitamin C is of great interest in the maintenance of genomic stability, and cancer prevention and treatment (Figure 2B).

### Activation of Tumor Suppressors and DNA Repair Enzymes by Vitamin C

CpG islands in the promoters of DNA damage response genes, such as the tumor suppressor *p16^*INK4a*^* and *p21*, are frequently hypermethylated in cancer (Roman-Gomez et al., 2002; Zhao et al., 2016; Ocker et al., 2019). The p16 and p21 proteins belong to a family of cyclin–dependent kinase (CDK) inhibitors, also known as CDKN2A and CDKN1A, respectively, that bind and inactivate CDKs to block cell cycle progression. Reactivation of their expression by vitamin C has been shown to induce senescence, apoptosis and halt proliferation of cancer cells. Vitamin C treatment of human skin and colon cancer cells increases 5hmC formation and reduces 5mC in the promoter CGIs of *p16*^*INK4a*^ and *p21* that correlates with upregulated expression (Lin et al., 2014; Gerecke et al., 2018). Vitamin C also upregulates p53 and p21 protein expression in other cancer cells, such as oral squamous cell (Zhou et al., 2020) and hepatocellular carcinoma (Lv et al., 2018) causing growth arrest and apoptosis, however, in these studies the effect on 5hmC/5mC levels at these gene loci was not measured.

The studies in cancer cells on the effect of vitamin C in regulating *p16*^*INK4a*^ and *p21* expression seem at odds with the observation in ESCs, iPSCs and tissue specific stem cell cultures in which vitamin C treatment silences the p16*^*INK*4*a*^/p19^*ARF*^* locus and is associated with reduced expression of p53 and p21 (He et al., 2008; Li et al., 2009; Esteban et al., 2010; Li Y. et al., 2016; Zhang P. et al., 2016). Furthermore, TET activity at promoter CGIs in stem cells may be counteracted by vitamin C-mediated co-activation of the H3K36 demethylase JHDM1b (KDM2B), which removes gene body H3K36me2/3 marks leading to p16*^*INK4a*^/p19^*ARF*^* gene silencing (He et al., 2008; Tzatsos et al., 2009). These different responses highlight the context specific effect of vitamin C in the regulation of senescence in normal stem cells compared to cancer cells. Overexpression of the reprogramming factors such as OCT4, SOX2, KLF4, and MYC increase ROS production by up to threefold compared to controls which could induce oxidative damage and trigger the premature senescence of non-cancerous stem cells (Krishnamurthy et al., 2004; Li et al., 2009). Vitamin C antioxidant effects can mitigate the high levels of ROS induced during reprogramming to prevent oxidative damage, thereby blocking ROS-induced senescence mechanisms (Krishnamurthy et al., 2004; Li et al., 2009).

Vitamin C treatment also induces the expression of a TET2-dependent gene expression signature in human leukemia cell lines and primary murine HSPCs involved in BER such as GADD45, PARP, and DNA glycosylases (Cimmino et al., 2017). The ability of vitamin C to drive increased BER activity could be a direct consequence of the increased need to actively remove and replace TET catalyzed oxidation products of mC in the DNA.

### Up-Regulation of Endogenous Retroviral Elements by Vitamin C

DNA methyltransferase inhibitors (DNMTis), such as 5-azacytidine and decitabine, are cytidine analogs used for the treatment of hematological malignancies by inducing global DNA hypomethylation (Hackanson et al., 2005; Shadduck et al., 2007; Santos et al., 2010). Restoring TET function by vitamin C administration, in combination with DNMTi therapy, may help erase DNA hypermethylation at tumor suppressor loci to promote differentiation and cell death. In addition, studies in a variety of cancers cells have shown that DNA hypomethylation by DNMTis causes the increased expression of endogenous retroviruses (ERVs) that mimic a viral infection and trigger an innate immune response leading to apoptosis (Chiappinelli et al., 2015; Roulois et al., 2015; Liu et al., 2016). Vitamin C was shown to synergize with DNMTi treatment in a TET2-dependent manner to increase 5hmC, and drive DNA hypomethylation to further increase ERV expression and enhance apoptosis of leukemia and solid tumor cell lines (Liu et al., 2016). Interestingly, ERVs in ESCs treated with vitamin C have been shown to acquire and retain high levels of 5hmC that do not become demethylated for several days, in contrast to the rapid and dynamic hydroxymethylation and demethylation induced at CGIs in promoters and TSS sites of hypermethylated pluripotency and blastocyst-like genes (Blaschke et al., 2013). This distinction may be a key factor in how vitamin C treatment, by promoting TET activity, can induce the expression of ERVs while preventing genomic instability that would be caused by a lack of 5mC or 5hmC at these retrotransposable elements (REs). REs are one of the two classes of TEs, which unlike the cut and paste mechanisms of DNA transposons, insert themselves by duplicating elements into a new genomic location via an RNA intermediate (Friedli and Trono, 2015; Chuong et al., 2016). In ESCs, TET2 has been shown to be recruited to ERV loci by an ERV RNA-binding protein Paraspeckle Component -1 (PSPC1) to regulate the expression of adjacent genes during embryonic development (Guallar et al., 2018). Maintenance of 5hmC levels at ERVs by vitamin C may therefore reduce the risk of insertional mutagenesis while exploiting these *cis*-regulatory sequences for transcriptional control of neighboring genes.

### Vitamin C-Mediated Effects on Telomere and Chromosomal Stability

Human telomeres shorten by 20–200 bp per cell division (Harley et al., 1990) and telomere length in circulating blood cells has been used as a biomarker of human aging (von Zglinicki and Martin-Ruiz, 2005). Higher intake of antioxidants by multivitamin supplementation or high vegetable intake is associated with increased telomere length in human studies (Xu et al., 2009; Marcon et al., 2012; Tsoukalas et al., 2019), however, direct evidence that vitamin C supplementation alone can suppress telomere attrition *in vivo* requires further study. Using *in vitro* cellular models derived from human iPSCs, vitamin C treatment has been shown to increase telomerase activity and the expression of genes encoding telomerase-related RNA and protein components that protect telomere stability (Wei et al., 2012; Kim et al., 2013). Patients with Werner Syndrome (WS) harbor a mutation in the *WRN* gene that leads to loss of telomere maintenance, premature aging and increased cancer rates (Burtner and Kennedy, 2010; Zhang et al., 2015). Vitamin C treatment of a human WS mesenchymal stem cell model was shown to slow down telomere loss and downregulate the senescence-inducing p16^*Ink4a*^ protein (Zhang et al., 2013, 2015). In ESCs, the protection and maintenance of telomere length by vitamin C has not yet been correlated directly to its role as a TET coactivator; however, given that mouse ESCs with TET deficiency exhibit shorter telomeres, chromosomal instability, sub-telomere DNA hypermethylation and reduced telomere recombination (Lu et al., 2014; Kafer et al., 2016; Lazzerini-Denchi and Sfeir, 2016; Yang et al., 2016), vitamin C most likely plays a direct role in TET-mediated telomere maintenance.

## Potential Roles for Vitamin C in Genomic Stability via Modulation of Additional α-KGDDs

Vitamin C can participate as a cofactor to enhance and maintain the activity of numerous other α-KGDD family members, such as histone lysine demethylases (KDMs), hypoxia inducible factor (HIF) prolyl hydroxylases, and AlkB homologs (ALKBHs), in addition to TET proteins, that in combination can influence genomic stability.

### Histone Lysine Demethylases (KDMs)

Vitamin C is required for the optimal activity and demethylation capacity of multiple KDMs that hydroxylate and remove mono-, di-, or trimethyl-lysines in histones (Klose et al., 2006; Tsukada et al., 2006). During somatic cell reprogramming, vitamin C-mediated removal of histone modifications such as H3K4me3 and H3K36me3, potentially by KDM5 and KDM2, respectively, can turn off the expression of genes, while demethylation of H3K9me2/me3 by members of the KDM3/4/7 family can remodel heterochromatin regions that can facilitate DNA hypomethylation at these otherwise silenced loci (Li et al., 2009; Tzatsos et al., 2009; Esteban et al., 2010; Huang et al., 2019). Histone modifications and KDMs have a wide variety of roles in regulating genomic stability that could potentially be influenced by vitamin C with different biological outcomes in either stem cells or cancer cells. Suppression of H3K9me2/me3 demethylase activity such as KDM3A (JMJD1A), KDM4C (JMJD2C), and KDM7C (PHF2) in tissue specific stem cell cultures and cancer cells can lead to premature senescence, increased DNA damage and genomic instability (Huang et al., 2019; Pappa et al., 2019; Fan et al., 2020). Vitamin C can coactivate KDM5B/C to demethylate H3K4me3, and KDM5B (JARID1B) has been shown to increase DNA DSB repair by recruiting factors Ku70 and BRCA1 in osteosarcoma (U2OS) cells (Li et al., 2014). A deficiency in KDM5B was shown to disengage the DNA repair process, promote spontaneous DNA damage, activate p53 signaling, and sensitize cells to genotoxic insults (Li et al., 2014). Demethylation of H3K4me3 by KDM5C (JARID1C) leads to heterochromatin formation, and renal cell carcinoma patients with *JARID1C* mutations exhibit genome-wide DNA hypomethylation, increased genomic rearrangements, and an overall worse prognosis (Rondinelli et al., 2015). In these settings, vitamin C could enhance KDM activity to prevent genomic instability. However, other KDM activity, such as overexpression of KDM2B (JHDM1B) and KDM4A (JHDM3A) that target H3K36 for demethylation could promote increased DNA damage through decreased HR repair (Pfister et al., 2014; Staberg et al., 2018).

### HIF Prolyl Hydroxylases

HIF-1 and HIF-2 are transcription factors induced in low oxygen (hypoxic) environments typical of tumor niches, and are repressed in normoxic conditions by prolyl hydroxylases (PHDs) (Rankin and Giaccia, 2016). Vitamin C has been shown to function as a direct cofactor of HIF PHDs (Osipyants et al., 2018) that catalyze the hydroxylation of HIF-1α (Appelhoff et al., 2004). HIF-1α is involved in microsatellite instability and mismatch repair deficiency in a colon cancer model (Koshiji et al., 2005) and induces transcriptional changes leading to the downregulation of several DNA damage repair genes in an oral squamous cell carcinoma model (Nakamura et al., 2018). Treatment with vitamin C could promote the degradation of HIF-1α by stimulating HIF prolyl hydroxylases that would restore DNA repair activity and remove HIF-1 α mediated protection from genomic instability (Knowles et al., 2003; Zhao et al., 2015). HIF stabilization is also associated with therapeutic resistance to DNA damage. Hypoxic mouse stromal cells are shown to be more resistant to irradiation than the same cells under normoxic conditions in a HIF-1 dependent manner (Calvo-Asensio et al., 2018), and a lung cancer model expressing a HIF-1-stabilizing micro RNA exhibits a hypoxic phenotype and increased radioresistance (Grosso et al., 2013). Inhibition of leukemia cell growth by vitamin C treatment also correlates with the downregulation of HIF-1α mRNA expression (Kawada et al., 2013). Vitamin C could therefore target the PHD family of α-KGDDs to maintain genomic stability and counteract hypoxic tumor conditions to influence disease progression or sensitivity to DNA damage.

### AlkB Homologs

In mammalian cells, homologues of the *E. coli* DNA dealkylation enzyme AlkB act as RNA demethylases, catalyzing the oxidative demethylation of *N*^6^-methyladenosine (m^6^A) (Fedeles et al., 2015). Nine mammalian homologues have been identified, ALKBH1-8 and the fat mass and obesity associated protein (FTO), with each displaying unique roles in genomic stability (Fedeles et al., 2015). ALKBH5 and FTO are the most well characterized of the AlkB homologs and their silencing or deletion reduces the expression of a number of HR and other repair genes in glioblastoma stem cells (Kowalski-Chauvel et al., 2020) and osteoblasts (Zhang et al., 2019), increasing their susceptibility to genotoxic damage. Interestingly, ALKBH2 and ALKBH3 have been shown biochemically to act as DNA repair enzymes that oxidize 5mC to generate 5hmC, 5fC, and 5caC, similarly to TET enzymes (Bian et al., 2019). However, in certain cancer cell models, ALKBH activity may promote survival or resistance to chemotherapy. *ALKBH2* knockdown sensitize lung cancer cells to cisplatin (Wu S.S. et al., 2011) and glioblastoma cells to temozolomide (Johannessen et al., 2013) and *ALKBH8*-knockout in mouse embryonic fibroblasts elevates ROS levels that could promote DNA damage (Endres et al., 2015). While vitamin C was shown to stimulate oxoglutarate turnover in *E. coli* AlkB (Welford et al., 2003), studies on the role of vitamin C in the regulation of mammalian ALKBH proteins are lacking. Given the recent confirmation that vitamin C binds directly to FTO (Wang et al., 2020) and the diverse roles of m^6^A in the regulation of gene expression in cancer (He et al., 2019) the role of vitamin C in m^6^A demethylation and the implication on genomic stability and cancer therapy requires further investigation.

## Metabolic Regulation of α-KGDDs, Genomic Instability, and Vitamin C

Isocitrate dehydrogenases (IDHs) are citric acid cycle enzymes responsible for making α-KG, and as such are inextricably linked with α-KGDD functional activity. Mutually exclusive IDH1 or IDH2 mutations are common in myeloid malignancies and gliomas, and cause the production of 2-hydroxyglutarate (2HG), an oncometabolite that impairs α-KGDD function by acting as a competitive inhibitor of α-KG binding (Dang et al., 2010; Han et al., 2020). 2HG inhibition in IDH-mutant AML patients impairs TET2 function that leads to loss of 5hmC and DNA hypermethylation (Figueroa et al., 2010a; Lu et al., 2012; Sasaki et al., 2012). 2HG can also cause H3K9 hypermethylation due to decreased KDM4B activity, which can block the recruitment of repair factors at DSB sites in the genome and reduce HR repair efficiency (Sulkowski et al., 2020). Vitamin C treatment, however, has been shown to reduce proliferation in an IDH-mutant leukemia model that was associated with demethylation at the loci of myeloid differentiating factors (Mingay et al., 2018), suggesting that it could potentially override the effect of 2-HG to activate α-KGDDs such as the TET demethylases.

## Vitamin C Deficiency and Effects on Cancer Progression

Vitamin C is an essential dietary micronutrient for humans, whereas other mammals, including mice, can synthesize vitamin C from glucose via the liver enzyme L-gulonolactone oxidase (GULO). GULO catalyzes the last step of ascorbate biosynthesis but is mutated and non-functional in humans (Linster and Van Schaftingen, 2007). Vitamin C is water soluble and optimal physiological plasma concentrations of approximately ∼70–80 μM can be sustained by the daily intake of 200 mg in the diet (Lindblad et al., 2013; Padayatty and Levine, 2016). Vitamin C is most well-known for its role in the prevention of scurvy, a disease caused by prolonged periods of low dietary vitamin C intake (<10 mg/day) that reduces plasma levels to below 11.4 μM, leading to insufficient collagen production that manifests with symptoms ranging from fever, confusion and depression, to internal bleeding (Prinzo, 1999; Schleicher et al., 2009; Padayatty and Levine, 2016).

While scurvy is now seen as a rare modern-day disease, patients with cancer are often markedly vitamin C-deficient (Mayland et al., 2005; Huijskens et al., 2016; Liu et al., 2016), and restoring or maintaining physiological levels has been shown to slow malignant cell growth (Campbell et al., 2015; Liu et al., 2016; Agathocleous et al., 2017). Plasma concentrations of ascorbate can differ up to ten-fold from person to person (Khaw et al., 2001) and in the United States, it is estimated that more than 7% of the population (>20 million people) are deficient in vitamin C (Schleicher et al., 2009). A study in the United Kingdom found that 25–46% of low income population and smokers exhibit deficient or depleted vitamin C plasma levels (Mosdol et al., 2008). Mild vitamin C deficiency may be underreported owing to its non-specific symptoms such as fatigue, irritability, dull aching pains, and weight loss (Prinzo, 1999; Schleicher et al., 2009). All-cause mortality decreases when vitamin C serum levels rise above 60 μM (Goyal et al., 2013; Wang et al., 2018), while in cancer patients, low serum vitamin C levels of <20–30 μM are frequently observed (Waldo and Zipf, 1955; Anthony and Schorah, 1982; Mayland et al., 2005; Carr et al., 2020). A study of leukocytes isolated from colon cancer patients showed decreased levels of 5mC and 5hmC that correlated with blood plasma ascorbate below 20 μM, while expression of TET genes was not significantly changed (Starczak et al., 2018). 5hmC/5mC ratios in the DNA of peripheral blood cells could potentially act as a biomarker of vitamin C status or bioavailability.

**FIGURE 3:**
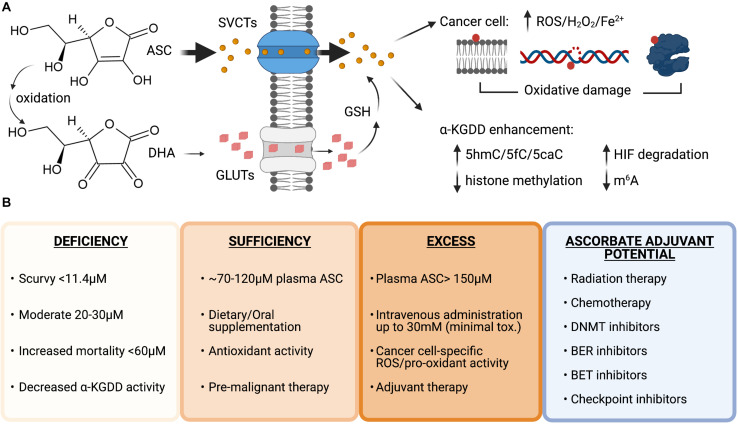
Vitamin C uptake and therapeutic potential as an anti-cancer agent. **(A)** Structures of ascorbate (ASC) and dehydroascorbate (DHA). ASC primarily enters cells through sodium-dependent vitamin C transporters (SVCTs) and can be transported in its oxidized form as DHA via glucose transporters (GLUTs) then reduced back to ASC by the antioxidant glutathione (GSH) once inside the cell. ASC can enhance the activity of α-ketoglutarate dependent dioxygenases (α-KGDD), and high doses can act as a pro-oxidant, creating increased levels of reactive oxygen species (ROS), H2O2 and increased redox active iron levels (Fe^2+^) that cause lipid, DNA, and protein oxidation in cancer cells. **(B)** Plasma ascorbate levels correlate with disease states, overall health, and therapeutic potential. Ascorbate deficiency is associated with increased all-cause mortality and lower α-KGDD activity. Oral supplementation can restore plasma levels to normal and allow vitamin C antioxidant properties to provide benefit as a pre-malignant therapy potentially lowering genomic instability and preventing transformation. High-dose intravenous administration of ASC can be used to generate millimolar (mM) plasma concentrations and generate ROS and pro-oxidant damage to cancer cells. Vitamin C has currently shown efficacy as an adjuvant for many cancer therapies in pre-clinical and clinical trials in combination with standard chemotherapy, DNA hypomethylating agents and targeted inhibitors: Carboplatin and Paclitaxel, Azacitidine, poly-adenosine diphosphate-ribose polymerase (PARP) inhibitors (Olaparib), immune checkpoint inhibitors (anti-PD1 and anti-CTLA4), and bromodomain and extraterminal domain (BET) inhibitors (JQ1). Figure created with BioRender.com.

Vitamin C is water soluble and transported across cellular membranes by sodium-dependent vitamin C transporters (SVCTs) and facilitative glucose transporters (GLUTs). SVCTs transport vitamin C directly, whereas GLUTs transport the oxidized form of vitamin C, dehydroascorbate (DHA), which is reduced to vitamin C inside cells by glutathione (GSH) (Figure 3A). Plasma levels of vitamin C are tightly controlled by two sodium-dependent vitamin C transporters (SVCTs), with SVCT1 being responsible for gastrointestinal absorption and renal reabsorption, and SVCT2, playing a primary role in whole body cellular uptake (Lindblad et al., 2013). Mouse models with genetic inactivation of the *Gulo* locus or *Svct1/2* knockout have been used to model cell-intrinsic or systemic dietary deficiency in vitamin C (Maeda et al., 2000; Sotiriou et al., 2002; Corpe et al., 2010). Supplementation of ascorbate in the drinking water at 3.3 g/L is sufficient to maintain normal 80 μM concentrations in the plasma of *Gul*o knockout mice; however, 0.33 g/L will reduce plasma concentrations to 30 μM (Schleicher et al., 2009; Kim et al., 2012). This model of dietary deficiency in mice causes hematopoietic defects that mimic the effect of TET2 deficiency, including pre-leukemic HSPC expansion and loss of 5hmC in the genome (Beamer et al., 2000; Sotiriou et al., 2002). Importantly, these effects could be reversed upon increased dietary vitamin C administration (Agathocleous et al., 2017). *Svct2* knockout in bone marrow cells expressing the AML oncogene *Flt3*^*ITD*^ has also been shown to accelerate leukemia progression in mice and exacerbate 5hmC loss in *Tet2* deficient HSCs, suggesting that vitamin C depletion could further impair the activity of other TET proteins in pre-leukemic cells (Agathocleous et al., 2017). Severe deficiency of both *Tet2* and T*et3*, is associated with >90% loss of 5hmC in HSPCs and causes the spontaneous accumulation of DSBs, marked by elevated and persistent yH2AX foci and rapid leukemia progression (An et al., 2015). Taken together, these findings suggest that vitamin C, by maintaining TET activity in HSPCs, provides protection from leukemia transformation.

## Effect of Vitamin C Treatment as an Anti-Cancer Therapy

Vitamin C supplementation is being explored as an adjuvant to many existing cancer therapies (Figure 3B). As cancer patients frequently present with decreased plasma vitamin C levels (Waldo and Zipf, 1955; Anthony and Schorah, 1982; Mayland et al., 2005; Carr et al., 2020) that are further decreased by chemotherapy (Mousseau et al., 2005; Carr et al., 2020), vitamin C treatment to restore normal amounts in the circulation as an adjuvant for standard chemotherapy could be of therapeutic benefit. The vast majority of patients are *TET2* haploinsufficient (Abdel-Wahab et al., 2009; Delhommeau et al., 2009; Kosmider et al., 2009; Langemeijer et al., 2009), suggesting that enhancing residual TET2 activity, or restoring the activity of functionally defective mutant TET2 proteins, could be a particularly viable therapeutic strategy for *TET2* deficient hematological malignancies. A recent study showed that oral supplementation of 500 mg/day of vitamin C in combination with DNMTi treatment in patients with myeloid malignancies was able to raise deficient vitamin C plasma levels to ∼100 μM concentration, increase 5hmC/5mC ratios and decrease global 5mC levels that were elevated at baseline in the peripheral blood cells (Gillberg et al., 2019). While previous clinical studies in advanced cancer patients given similar oral doses did not show any benefit (Creagan et al., 1979; Moertel et al., 1985), long-term oral vitamin C supplementation may have important clinical implications for pre-malignant and low-risk patients with mutation in specific epigenetic regulators such as *TET2* and should be investigated further.

### High-Dose Vitamin C Pro-oxidant Roles in Cancer Cells

Maximal plasma concentrations from oral vitamin C doses above 500 mg/day do not exceed 150 μM, due to homeostatic down-regulation of vitamin C transporters in enterocytes and kidney cells, leading to reduced absorption and increased urinary excretion (Young et al., 2015). High-dose vitamin C (up to 100 g) administered by intravenous (IV) infusion can bypass the limited oral bioavailability without reported toxicity, and raise plasma concentrations to high millimolar levels that remain above 100 μM for up to 6 h (Padayatty et al., 2004). An intriguing effect of high-dose vitamin C treatment in cancer cells is its ability to create ROS and act as a pro-oxidant.

Vitamin C in the form of ascorbate, under normal growth conditions, behaves as an anti-oxidant that donates electrons to quench damage-inducing free radicals, becoming a relatively stable ascorbate radical while to protecting cells from lipid, protein, and DNA oxidation (Padayatty et al., 2003). In contrast to the effect vitamin C has in reprogramming and stem cell cultures, which is to reduce ROS, increase proliferation and protect cells from apoptosis, vitamin C treatment of cancer cells correlates with an increase in ROS-mediated oxidative stress and reduced viability (Chen et al., 2008; Noguera et al., 2017; Ghanem et al., 2020; Wu et al., 2020; Zhou et al., 2020). Moreover, it has been demonstrated that while diverse cancer cell types are sensitive to low- and sub-millimolar doses of vitamin C, even with high millimolar doses, limited cytotoxicity is observed in normal tissue (Chen et al., 2008; Noguera et al., 2017). This points to a unique synthetic lethality of cancer cells to high-dose vitamin C treatment that can be exploited for cancer therapy. In a Burkitt’s lymphoma model, toxicity to ascorbate was associated with ROS-induced peroxide formation that could be abrogated by catalase treatment (Chen et al., 2005). Follow up work by the same group showed elevated ascorbate radical and peroxide levels *in vivo* in the microenvironment of three separate models where vitamin C reduced tumor burden in mice (Chen et al., 2008). Another study showed mitochondrial hyperpolarization associated with high-dose ascorbate and ROS production (Ghanem et al., 2020).

The pro-oxidant effects of vitamin C in these studies has been attributed to excess uptake and formation of redox active iron. In a series of reactions, ascorbate can reduce ferric (Fe^3+^) iron to ferrous (Fe^2+^), which can then be returned to the ferric state after reducing O_2_ to superoxide. Superoxide reacts with itself and protons to generate hydrogen peroxide and O_2_, and the hydrogen peroxide can be reduced by Fe^2+^ in a Fenton reaction to form hydroxide radicals that can create the pro-oxidant killing effects observed in cancer cells (Du et al., 2012). Another possibility is that high-dose vitamin C can exhaust the primary cellular antioxidant GSH when taken up in higher amounts by more metabolically active cells, thus rendering cancer cells more vulnerable to oxidative stress. In this capacity, high-dose vitamin C was shown to be selectively toxic to KRAS or BRAF mutant colorectal cancer cells due to increased cellular uptake of oxidized vitamin C (DHA) through upregulated GLUT transporters, which led to GSH depletion and lethal levels of ROS (Yun et al., 2015).

### Combination Cancer Therapies With Vitamin C Treatment

Multiple cellular and animal models and recent clinical trials have shown that high-dose vitamin C treatment can reduce cancer cell viability and improve treatment outcome in combination therapies. In patients with ovarian cancer, high-dose intravenous vitamin C (IVC) administered in combination with carboplatin and paclitaxel enhanced chemosensitivity and reduced adverse side effects of chemotherapy (Ma et al., 2014). Glioblastoma patients receiving IVC in combination with radiation therapy and temozolomide have also shown improved survival compared to previous studies (Schoenfeld et al., 2017). Another notable adjuvant use of vitamin C was with PARP inhibitor treatment for a small group of patients with HR-deficient advanced metastatic cancers (primarily BRCA mutations) where partial or complete responses were observed in all eight patients (Demiray, 2020).

High-dose parenteral vitamin C treatment can be modeled in mice via IP administration, where 4 g/Kg delivered IP can induce mM concentrations in plasma similar to pharmacokinetic studies in patients treated with high-dose IVC (Padayatty et al., 2004; Yun et al., 2015). Vitamin C treatment of *Tet2*-deficient mouse HSPCs and patient-derived AML leads to increased 5hmC formation, DNA hypomethylation and a block in aberrant self-renewal *in vitro* with suppression of disease progression *in vivo* (Cimmino et al., 2017). High-dose vitamin C also enhances immune checkpoint inhibitor therapy, that in animals models was shown to be curative when used to treat tumors with high mutational burdens such as mismatch repair–deficient (MMRd) or microsatellite instable (MSI) breast and colorectal mouse tumors that otherwise would be unresponsive to single immune checkpoint inhibitors (Magri et al., 2020). MSI cancers exhibit microsatellite DNA hypermethylation and accumulate 5mC mutations in CpG in the absence MMR that has been attributed to methylation-associated repair deficiencies (Ling et al., 2010; Poulos et al., 2017). While a role for TET mediated hypomethylation in microsatellite stability was not assessed, this may be one mechanism by which vitamin C treatment could play a role in MMR-deficient cancer treatment. Importantly the therapeutic efficacy of vitamin C in combination with checkpoint inhibitors was dependent on a functional immune system, suggesting that tumor cell-intrinsic and immune microenvironmental effects of vitamin C can work together to slow tumor growth.

Multiple studies continue to identify new potential combination cancer treatment strategies with vitamin C. In cellular and preclinical studies, high-dose vitamin C enhances the sensitivity of hematological cell malignancies to arsenic trioxide (Huijskens et al., 2016; Noguera et al., 2017) and increases chemosensitivity and radiosensitivity of various solid tumor cells including ovarian (Ma et al., 2014), pancreatic (Du et al., 2015) glioblastoma and non-small cell lung carcinoma cells (Schoenfeld et al., 2017). Vitamin C has also been shown to sensitize melanoma cells to bromodomain inhibitors (Mustafi et al., 2018). These studies argued in favor of combination therapies based primarily on the mechanism of oxidative stress generated by high-dose ascorbate, that could increase cancer cell susceptibility to standard therapies by exacerbating DNA damage. The effect of vitamin C as a co-factor of α-KGDDs and specifically as a regulator of DNA demethylation via increased TET function were not investigated. However, it has been shown that TET-mediated DNA oxidation induced by vitamin C can create a synthetic lethality, where AML cells being forced to undergo active DNA demethylation renders them hypersensitive to PARP inhibition (Cimmino et al., 2017). In this study, vitamin C treatment increases the expression of genes involved in BER that may be a response to the increased oxidation of 5mC and in combination with the PARP inhibitor, Olaparib, enhances the killing of human AML cells greater than either agent alone (Cimmino et al., 2017). Future studies on the use of vitamin C as a therapeutic agent would benefit from the inclusion of additional correlative studies such as DNA methylation changes, oxidized mC formation or even histone and RNA methylation levels in order to fully appreciate the cell-intrinsic or microenvironmental epigenetic biomarkers of its anti-cancer efficacy.

## Conclusion

Given the numerous roles for vitamin C in biological processes that maintain and influence genomic stability, it is no surprise that vitamin C continues to be explored for potential anticancer activity. From the studies by Cameron and Pauling in the 1970s (Cameron and Pauling, 1976, 1978) to the present day, there is an ever-expanding volume of research attempting to elucidate the true and, more relevantly, meaningful roles of vitamin C in disease etiology, treatment, and prevention. But important consideration must be given to robust data collection and interpretation, especially where clinical samples are involved (Lykkesfeldt, 2020). Considering vitamin C’s accessibility and prevalence as a dietary nutrient, synthesizing meaningful conclusions on the role of vitamin C in cancer prevention by oral supplementation will require large population studies. However, vitamin C’s role as an anti-tumor agent that can restore DNA damage and repair signaling processes at high doses appears to go beyond just regulating the oxidation state of the cell. Vitamin C has also been shown to influence many enzymes within the Fe^2+^ and α-KGDD superfamily that can work together to maintain genomic stability beyond the direct effect of vitamin C in TET-mediated modulation of cytosine modification and turnover in the genome. The role of vitamin C as an epigenetic regulator will have context specific effects that depend on the cancer cell lineage. The strong association of DNA hypermethylation coupled with the frequent mutation of *TET2* in hematological malignancies suggest that vitamin C treatment could be a targeted therapy for these patients. Future studies into how vitamin C can maintain the function of the diverse α-KGDDs known to have direct and indirect roles in the maintenance of genomic stability will allow us to fully understand the effect of this essential vitamin in the etiology, prevention, and treatment of cancer.

## Author Contributions

JB and TL contributed equally in the writing and organization of the manuscript. SM contributed to the writing of the manuscript. LC supervised and edited the manuscript. All the authors contributed to the article and approved the submitted version.

## Conflict of Interest

The authors declare that the research was conducted in the absence of any commercial or financial relationships that could be construed as a potential conflict of interest.
